# Weight Loss Results and Compliance with Follow-up after Bariatric Surgery

**DOI:** 10.1007/s11695-021-05450-6

**Published:** 2021-05-08

**Authors:** Beata M. M. Reiber, Anna-Marie R. Leemeyer, Marjolein J. M. Bremer, Maurits de Brauw, Sjoerd C. Bruin

**Affiliations:** 1 Department of Gastro-Intestinal Surgery, Amsterdam University Medical Center, location VUmc, De Boelelaan 1117, 1081 HV Amsterdam, the Netherlands; 2 Department of Intensive Care Medicine, Leiden University Medical Center, Leiden, the Netherlands; 3 Department of Emergency Medicine, Dijklander Hospital, Hoorn, the Netherlands; 4 Department of Bariatric Surgery, Spaarne Gasthuis, Haarlem, the Netherlands

**Keywords:** Laparoscopic Roux-en-Y gastric bypass, Midterm follow-up, Adherence to follow-up, Loss to follow-up, Postoperative weight loss

## Abstract

**Purpose:**

The importance of follow-up (FU) for midterm weight loss (WL) after bariatric surgery is controversial. Compliance to this FU remains challenging. Several risk factors for loss to FU (LtFU) have been mentioned. The aim was therefore to evaluate the association between WL and LtFU 3 to 5 years postoperatively and to identify risk factors for LtFU.

**Materials and Methods:**

A single-center cross-sectional study in the Netherlands. Between June and October 2018, patients scheduled for a 3-, 4-, or 5-year FU appointment were included into two groups: compliant (to their scheduled appointment and overall maximally 1 missed appointment) and non-compliant (missed the scheduled appointment and at least 1 overall). Baseline, surgical, and FU characteristics were collected and a questionnaire concerning socio-economic factors.

**Results:**

In total, 217 patients in the compliant group and 181 in the non-compliant group were included with a median body mass index at baseline of 42.0 and 42.9 respectively. Eighty-eight percent underwent a laparoscopic Roux-en-Y gastric bypass. The median percentage total weight loss for the compliant and non-compliant groups was 30.7% versus 28.9% at 3, 29.3% versus 30.2% at 4, and 29.6% versus 29.9% at 5 years respectively, all *p*>0.05. Age, persistent comorbidities and vitamin deficiencies, a yearly salary <20,000 euro, no health insurance coverage, and not understanding the importance of FU were risk factors for LtFU.

**Conclusion:**

Three to 5 years postoperatively, there is no association between LtFU and WL. The compliant group demonstrated more comorbidities and vitamin deficiencies. Younger age, not understanding the importance of FU, and financial challenges were risk factors for LtFU.

**Graphical abstract:**

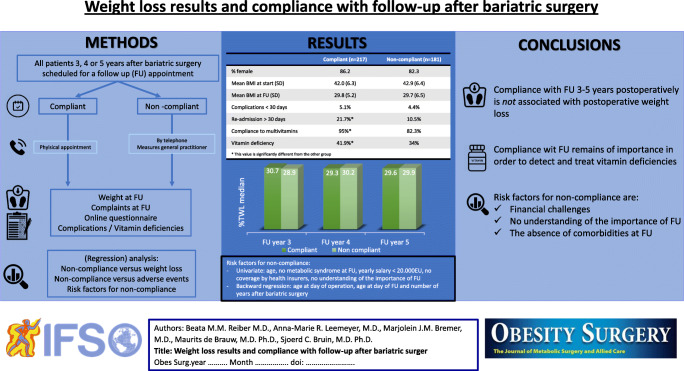

## Introduction

Over the past decades, bariatric surgery has become increasingly important in treating morbid obesity and has proven to not only result in weight loss (WL) but also effectively reduce comorbidities resulting from metabolic syndrome [1–3]. Multiple authors have emphasized the importance of adherence to follow-up (FU) after bariatric surgery; arguments are the timely recognition of late complications and vitamin deficiencies possibly leading to irreversible (neurological) disorders [4, 5]. In addition, several authors have pointed out the association between loss to FU and WL [6–8]. Compliance to this FU however remains extremely challenging [9, 10]. Multiple factors have been suggested as risk factors for this attrition among which distance to travel to the clinic, younger age, unemployment, and financial factors as well as psychological issues [7, 11–16]. Most studies have been carried out in the USA, which is a country that faces different challenges concerning health insurance, travel distances, and social-economic factors than the Netherlands. Moreover, most of these studies involve short-term FU only.

The efficacy and hence the necessity of clinical treatment and FU appointments are subject to debate. This appears specifically important in bariatric surgery as the burden of disease continues to rise and consequent logistical challenges can be foreseen. Therefore, the primary aim of this study was to explore the possible association between compliance to follow-up appointments at 3 to 5 years after bariatric surgery and postoperative WL. The secondary aim was to identify a group of patients at risk for loss to follow-up.

## Methods

### Study Population

This cross-sectional study was carried out in a bariatric center of excellence in the Netherlands. All patients after a primary laparoscopic Roux-en-Y gastric bypass (LRYGB) or a laparoscopic adjustable band removal and laparoscopic gastric bypass (redo LRYGB) scheduled for a 3-, 4-, or 5-year FU appointment between June and October 2018 were included. Patients were excluded when refusing to participate in this study. All patients were eligible for bariatric surgery according to the IFSO criteria. Informed consent was obtained both preoperatively and at follow-up. The study was approved by the Medical Ethical Committee.

Patients were divided into 2 groups: the compliant and non-compliant group. Patients included in the compliant group were allowed to miss one appointment during their entire FU period. Patients that missed the scheduled appointment *and* at least 1 other appointment were included in the non-compliant group.

### Data collection at Baseline

Of all patients the following details were collected:

- *Baseline characteristics*: gender, age at operation and follow-up date, weight and height at the day of operation, presence of metabolic syndrome, and comorbidities at intake and FU.
- *Surgical characteristics*: type of operation, postoperative complications within 30 days according to the Clavien-Dindo classification (CDC), and readmission and reoperation during the entire follow-up period in any hospital or clinic.

### Data Collection at Follow-up

FU was conducted in compliance with the current guidelines: 2 and 6 weeks postoperatively and every 3 months during the first year, twice during the second year, and annually after that. The total number of missed appointments during the entire FU period was collected.

The following details were collected at the FU appointment (compliant) or by phone (non-compliant) during the study period:

- *Symptoms*: intake-, dumping-, stool-related pain, abdominal and general pain, or mental complaints.
- *Exercise*: any complaints of fatigue, if patients were active for at least 30 min a day and if they conducted a sport—and if so, which and how often.
- *Metabolic comorbidities and vitamin deficiencies*: both compliance to the usage of multivitamins and medication taken and/or necessary for metabolic comorbidities were evaluated, data of concomitant visits to medical doctors treating comorbidities, and vitamin deficiencies were collected with the consent of the patients.
- *Reoperations and readmissions > 30 days after bariatric surgery*: patients were asked if they had been readmitted or reoperated elsewhere after bariatric surgery. If this was the case and patients consented, surgical details were collected.
- *Weight specifics*: current weight.
- *Questionnaire*: based on the available literature, an online questionnaire was developed to evaluate patient-related risk factors for non-compliance to FU appointments. These factors include socio-economic status such as marital status, employment- and insurance state, and the perception of the importance of FU by the patient. The English version of this questionnaire is included in the Appendix.

### Weight Outcomes

Postoperative percentage total weight loss (%TWL) was defined as ((weight at admission – follow-up weight) / weight at admission) × 100%. Sufficient %TWL was defined as %TWL at FU over 20%. Body mass index (BMI) was calculated as weight (kg)/height^2^ (m). Percentage excess BMI loss (%EBMIL) was calculated as ((BMI at admission – follow-up BMI)) / (BMI at admission – 25) × 100.

### Surgical Details

Preoperatively patients were demanded to lose 6 kilograms. If they did not reach this weight, the operation was postponed. Patients were admitted at the day of the operation. A LRYGB was carried out as a gold standard. The biliary limb length measured 50 cm and the alimentary limb 150 cm. The gastrojejunostomy (GJ) was created in an antecolic antegastric fashion with a side to side 30-mm stapled anastomosis. The remaining defect was closed with a V-loc suture. The jejuno-jejunostomy was created side to side 60 mm stapled with the closure of the remaining defect with a 60-mm stapler.

### Statistical Analysis

All baseline and operative characteristics are presented for the entire population and per group. Dichotomous outcomes are presented as the number of events with corresponding percentages and compared using the chi-square test or Fisher’s exact test according to group size. Continuous data are presented as means with standard deviation (SD) or median and interquartile range (IQR) and compared by the Student *T*-test, Mann-Whitney *U*-test, or Kruskal Wallis test according to normality and number of groups. Association of %TWL and compliance/non-compliance was evaluated for the entire population as well as for each follow-up year separately both through non-parametric tests as well as univariate analysis corrected for abnormal distribution of %TWL. Possible risk factors both %TWL and non-compliance to FU were identified through univariate analysis. Factors demonstrating *p<*0.2 were included in the linear and logistic backward regression analysis for %TWL and non-compliance respectively. The goodness of fit of the models was tested by *R*^2^/*R*^2^ adjusted and the Hosmer and Lemeshow test for respectively %TWL and non-compliance.

## Results

### Baseline Characteristics

Three hundred ninety-eight patients were included out of the 464 eligible patients: 217 in the compliant group and 181 in the non-compliant group. Of the 66 excluded patients, 16 had moved abroad and 50 did not consent to participate. The percentage and reasons of excluded patients were equally distributed over both groups. Of the included patients, 77 (19%) did not fill out the online questionnaire; these were evenly distributed across the groups. The percentage of non-compliant patients increased per FU year: 15% in year 3, 32% in year 4, and 53% in year 5. The median FU of the entire group was 4 years. The baseline characteristics and details concerning metabolic comorbidities are summarized in Table 1.

**Table 1:** Baseline characteristics

	All (*n*=398 )	Compliant (*n*= 217 )	Non-compliant (*n*= 181)	*p* value
Female; (%)	84.4	86.2	82.3	0.179
Age at intake; median (IQR)	45 (14)	45 (14)	45 (13)	0.884
Metabolic syndrome at intake; *n* (%)	127 (31.9)	91 (41.9)	36 (19.9)	0.000
Persistent at FU; *n* (%)	41 (10.3)	38 (17.5)	3 (1.7)	0.000
Diabetes Mellitus at intake; *n* (%)	56 (14.1)	37 (17.1)	19 (10.5)	0.001
Persistent at FU; *n* (%)	16 (4)	11 (5.1)	5 (2.8)	0.000
Hypertension at intake; *n* (%)	99 (24.9)	69 (31.8)	30 (16.6)	0.000
Persistent at FU; *n* (%)	31 (7.8)	29 (13.4)	2 (1.1)	0.000
OSAS at intake; *n* (%)	17 (4.3)	11 (5)	6 (3.3)	0.219
Persistent at FU; *n* (%)	9 (2.3)	7 (3.2)	2 (1.1)	0.220
Hypercholesterolemia at intake; *n* (%)	20 (5)	12 (5.5)	8 (4.4)	0.272
Persistent at FU; *n* (%)	14 (3.5)	10 (4.6)	4 (2.2)	0.226
Number of missed appointments median; (IQR)	1 (3)	0 (1)	3 (2)	0.000

*FU*, follow-up; *IQR*, interquartile range; *OSAS*, obstructive sleep apnea syndrome

### Surgical Characteristics

In both groups, LRYGB was performed in the majority of patients: 88.9% in the compliant and 87.8% in the non-compliant group. The remaining patients underwent a redo LRYGB. Complications within 30 days were evenly distributed between the compliant and non-compliant group: overall 5.1% vs 4.4% respectively (*p*=0.476) and CDC IIIb 2.3% vs 2.7% respectively (*p* 0.395). No complications of CDC IV/V occurred in either group. Details per group concerning reoperations and readmissions more than 30 days after bariatric surgery are shown in Tables 2 and 3.

**Table 2:** Reoperations > 30 days postoperatively

	Compliant (*n*=217)	Non-compliant (*n*=181)	*p* value
Reoperation > 30 days; *n* (%)	56 (25.8)	36 (19.9)	0.101
Laparoscopic cholecystectomy; *n* (%)	24 (11.1)	20 (11)	0.322
Laparoscopic internal herniation; *n* (%)	8 (3.7)	2 (1.1)	0.203
Diagnostic laparoscopy; *n* (%)	7 (3.2)	2 (1.1)	0.267
Trocar herniation; *n* (%)	0 (0)	2 (1.1)	0.118
ACNES; *n* (%)	1 (0.5)	0 (0)	0.369
Plastic surgery; *n* (%)	14 (6.5)	7 (3.9)	0.395
Other; *n* (%)	2 (0.9)	3 (1.7)	0.195
More than 1 reoperation > 30 days; *n* (%)	10 (4.6)	7 (3.9)	0.360

*ACNES*, anterior cutaneous entrapment syndrome

**Table 3:** Readmissions > 30 days postoperatively

	Compliant (*n*=217)	Non-compliant (*n*=181)	*p* value
Readmission > 30 days; *n* (%)	47 (21.7)	19 (10.5)	0.002
Ferinject; *n* (%)	27 (12.4)	4 (2.2)	0.013
Marginal ulcer; *n* (%)	10 (4.6)	2 (1.5)	0.007
Observation pain e.c.i.; *n* (%)	6 (2.8)	9 (5)	0.000
Observation dysphagia; *n* (%)	2 (0.9)	3 (1.7)	0.002
Observation general symptoms; *n* (%)	2 (0.9)	0 (0)	0.005
Stenosis GJ with stent placement; *n* (%)	0 (0)	1 (0.6)	0.001

e.c.i., unknown cause; *GJ*, gastrojejunostomy

### Weight-Related Results

**Fig. 1:**
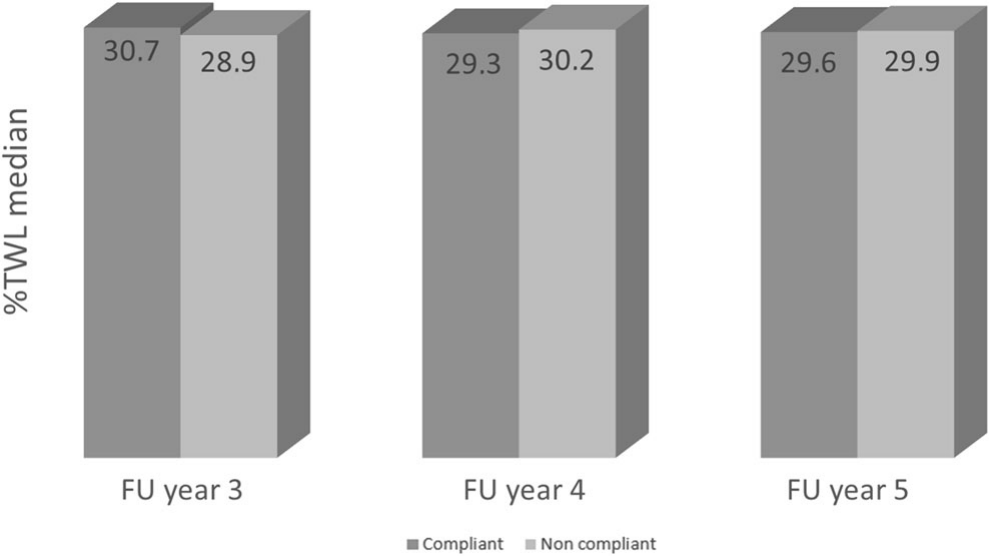
Median %TWL for each group per follow-up year. Abbreviations: FU, follow-up; %TWL, percentage total weight loss

Table 4 demonstrates the %TWL and %EBMIL at baseline and FU. Figure 1 shows the median %TWL for each group per FU year. There were no statistically significant differences between the groups both at baseline or FU (*p*>0.5). No statistical association was uncovered performing univariate, linear, or binary (regression) analysis including %TWL, sufficient versus non-sufficient %TWL, the number of missed appointments, and compliant versus non-compliant for the group total nor for each year separately. Stepwise regression analysis for the entire population produced a model predicting %TWL with an R square and adjusted R square of 37.6 and 33.1% respectively. The following factors were included in the model (B; lower—higher bound confidence interval): a higher age at operation and FU date (2.7; 1.40–3.98), female sex (3.43; 0.57–6.28), non-compliance to FU (− 7.55; − 11.7 to 3.41), higher start BMI (0.62; 0.42–0.84), no reoperation > 30 days (− 4.4 (− 6.68 to − 2.1), number of years after operation (2.27; 0.79–3.75), metabolic syndrome present at FU (3.11; 0.2–6.21), hypertension at intake (− 3.6; − 6.8 to 1.11), diabetes mellitus at intake (− 2.99; − 0.75 to 6.05), no general or mental complaints (4.2; 2.19–6.24 and 2.05; 0.24–4.81 respectively), absence of fatigue (2.05; − 0.34 to 4.43), employment (2.23; − 0.16 to 4.62), and smoking (− 5.77; − 8.26 to 3.27).

**Table 4:** Weight specifics

	Compliant (*n*= 217 )	Non-compliant (*n*= 181)	*p* value
BMI at operation day mean (SD)	42.0 (6.3)	42.9 (6.4)	0.107
BMI at follow-up mean (SD)	29.8 (5.2)	29.7 (6.5)	0.797
Change in BMI median (IQR)	− 12.8 (6.5)	− 12.9 (6.3)	0.737
%EBMIL mean (SD)	73.6 (20.8)	72.7 (24.4)	0.674
%TWL median (IQR)	30 (12.6)	30 (13.9)	0.956
Sufficient %TWL (>20%)	87.6%	87.4%	0.536

*BMI*, body mass index; *%EBMIL*, percentage excess BMI loss; *IQR*, interquartile range; *SD*, standard deviation; *%TWL*, percentage total weight loss

### Adherence to Multivitamin Supplements

At FU, 91 compliant patients were treated for vitamin deficiencies (42%) versus 63 (35%) non-compliant patients (*p*=0.036). Seventy-three (80%) compliant patients with a deficiency reported symptoms of fatigue, agitation, and depressive feelings. No neurological symptoms were reported. Deficiencies were as follows: 56 (62%) iron deficiency, 15 (16%) B_12_ deficiency, 10 (11%) calcium deficiency, 7 (8%) folic acid deficiency, 2 (2%) vitamin D deficiency, and 1 (1%) vitamin B_1_ deficiency.

All non-compliant patients with a deficiency reported symptoms of fatigue and depression. No neurological symptoms were reported by any of the patients during follow-up phonecall. Deficiencies were as follows: 13 (21%) iron deficiency, 35 (56%) B_12_ deficiency, 3 (4%) calcium deficiency, 11 (18%) vitamin D deficiency, and 1 (2%) vitamin B_1_ deficiency. Treatment of severe iron deficiencies was by parenteral iron suppletion in 27 (28.8%) compliant and 4 (6.5%) non-compliant patients. All other deficiencies were treated according to national protocol by oral suppletion or parenteral suppletion of vitamin B_12_. At the time of FU at the compliant group, 91% of patients were successfully treated; the remaining 9% were being treated at the time of follow-up of this study. In the compliant group, the success rate of deficiency treatment was 98%, and the remaining 2% was being treated at the time of follow-up of this study. In total, 207 (95%) of compliant patients were compliant to mandatory multivitamin usage versus 149 (82.3%) of the non-compliant patients (*p*=0.000). Eighty-eight (42.5%) patients compliant to both FU and multivitamins developed a deficiency versus 55 (37%) non-compliant patients that were compliant to the multivitamins (*p*>0.05).

### Risk Factors for Non-compliance

Table 5 shows the factors included in the regression analysis (i.e., baseline, surgical, and FU characteristics including the questionnaire), identified through univariate analysis. Stepwise regression analysis produced a model predicting non-compliance with a goodness of fit *p* value of 0.985 and a Cox and Snell *R*^2^ of 62%; age at the day of operation (B −4.348, OR 77.32), age at the day of FU (B −4.359, OR 0.013), and the number of years after bariatric surgery (B 4.419, OR 82.976).

**Table 5:** Factors independently associated with (non) compliance to follow-up

Factors associated with *p* < 0.05	Factors associated with *p* < 0.2
Age at the day of operation *and* day of FU	Gender
Metabolic syndrome at the day of FU	Readmission > 30 days after bariatric surgery
Diabetes mellitus at the day of FU	%TWL (corrected for abnormal distribution)
Hypertension at the day of FU	Presence of any abdominal complaints
Compliance to multivitamins	Presence of fatigue
Presence of vitamin deficiencies	Presence of any complaints
Number of years after bariatric surgery	
Yearly salary less than EU 20,000	
Coverage of FU costs by the health insurer	
Understanding of the importance of FU	

*FU*, follow-up; *%TWL*, percentage total weight loss

## Discussion

Several conclusions can be drawn from this study. First of all, 3 to 5 years after bariatric surgery, there is no association between compliance to follow-up and total weight loss. Secondly, the patients compliant to follow-up demonstrate both more persistent comorbidities and vitamin deficiencies as well as a higher compliance rate to prescribed multivitamins. Thirdly, factors independently associated with non-compliance were younger age and no understanding of the importance of follow-up and financial challenges.

Previous studies have mostly described a significant association between compliance to follow-up and postoperative weight loss. But these studies analyzed the results at one year postoperatively only [6, 17]. Other studies have shown that loss to follow-up increases dramatically over the years postoperatively and is naturally best at 1 year FU [18]. Lujan et al. recently published data including patients up to 3–5 years after LRYGB and SG separately. Interestingly, the compliant LRYGB group demonstrated a significantly higher percentage of excess weight loss (%EWL) compared to the non-compliant group [19]. These differences however did not persist when %TWL was compared. This is probably explained by the fact that the non-compliant group had a significantly higher BMI preoperatively with a difference with the compliant group of nearly 9 points. As previously suggested, %EWL but not %TWL is heavily influenced by the preoperative BMI of patients in favor of patients with a lower BMI [20, 21]. It is therefore remarkable that Lujan et al. based their conclusion on %EWL only. In the current study, both groups were more comparable than previous studies; there was no difference in preoperative BMI, nor in postoperative %TWL or %EBMIL. In a post hoc analysis, %EWL did not differ between the study groups either.

Several patient-related factors were identified as important for compliance to FU. Age has repeatedly been reported to be positively associated with compliance [11–13]. This was reaffirmed by this study and may well be explained by both the higher importance of personal health in older patients and a more stable household [14]. It may be of no surprise that compliant patients exhibit significantly more comorbidities as well as more readmissions. The explanation may lay in the extension of the understanding of the importance of FU; one could hypothesize that patients with comorbidities are more aware of their personal health—or the lack of it. In addition, compliant patients with comorbidities may already be used to intensive FU appointments whereas non-compliant patients without comorbidities feel healthy and may therefore experience little reason to “waste time” going to the hospital for just a check-up. A more pragmatic reason for the association of persisting comorbidities and the compliance to FU is probably the fact that most patients of our study group went to the same hospital for both FU appointments for their comorbidities and the bariatric FU. This might explain the difference between the current and a previous study that showed an inverse relationship between comorbidities and compliance to FU [14]; patients of that study were thought to visit different clinics and hospitals for their comorbidities and therefore not show up to the bariatric center. Previous studies have already shown that the presence of comorbidities is not associated with postoperative weight loss, which was reaffirmed in the current study [22].

In the univariate analysis, independent factors such as a yearly salary of less than 20,000 euro as well the absence of coverage of the FU costs by the health insurer were identified. Moreover, the non-compliant group in this study saw significantly less value of the FU appointments and was significantly less compliant to the multivitamins. This is similar to previous studies [11–13, 15]. All together, this strengthens the hypothesis that loss to FU in bariatric surgery seems to be a problem of motivation mostly and financial issues to a lesser extent.

It is remarkable that 37–42.5% of patients who reported to be compliant to multivitamins developed a vitamin deficiency. This might be due to the possibility that not all of these self-reported compliant patients actually do take their multivitamins daily. However, it has been suggested that the effectiveness of optimized supplements has higher effectiveness than standard supplements [23].

The question now remains how important adherence to FU truly is if it does not make a difference for postoperative weight loss. Both groups in this study demonstrated similar rates of reoperations. Due to a low incidence of internal herniation and ischemic bowel consequently or complicated cholecystolithiasis, we were not able to demonstrate significant differences of late severe complications between the groups. It is possible that nutrient deficiencies would become more apparent and clinically significant at FU after 5 years [5]. In other words, the possible danger to the health of the patient that is not compliant to FU and therefore subject to patient delay could not be confirmed nor denied. However, the non-compliant group in this study does demonstrate previously established risk factors for hematological, metabolic, and especially neurological disorders which are not always reversible, such as poor compliance with vitamin supplement intake and regular FU visits [4].

This study is limited by the retrospective collection of the baseline characteristics, despite having collected follow-up data prospectively. Collecting FU data via telephone in the non-compliant group may have affected the results. This risk of reporting bias of patients was attempted to be minimized by verifying the weight with the GP. As for the non-compliant group concerning vitamin deficiencies particularly, an underestimation of the percentage of patients developing a vitamin deficiency is certain as these patients were only tested when they reported complaints to their GP. In addition, it should be noted that WL after bariatric surgery has previously shown to be a complex and multifactorial phenomenon. This is clearly reflected in the results of the backward regression analysis on %TWL and its low adjusted R squared and the number of included factors. Hypothesizing that compliance to FU would be an isolated risk factor for disappointing WL is therefore perhaps too simplistic. As baseline characteristics were similar, risk of bias by these factors was attempted to remain limited.

## Conclusion

Compliance to midterm (3–5 years) follow-up is not associated with weight loss after bariatric surgery. Based on this study, it seems that patient-reported motivational and financial issues as well as the absence of comorbidities are related to non-compliance. Follow-up remains necessary as vitamin deficiencies despite compliance to multivitamin supplements and consequent possible complications are of persistent worry. In order to increase the adherence to follow-up, we should inform the patients more strenuously about the importance of FU. In addition, to improve the follow-up rate, we suggest a role of the health insurers here; annual FU appointments should be included in the covered costs. The question that remains to be answered is whether the annual mid- and long-term follow-up appointments can be replaced by the general practitioner.

## Appendix. Online questionnaire

According to our data you have / have not missed several appointments at our outpatient clinic. To understand why you have / have not showed up for the appointments, we would like to ask you a few questions.

1. Are you contact with the result of the operation?
  - Yes
  - No
2. Currently I….(multiple choice):
  - Have a few symptoms or complaints
  - Have a lot of symptoms or complaints
  - Have lost too much weight
  - Have lost too little weight
  - None of the above
3. Do you feel that the annual follow up appointments are important?
  - Yes
  - No, because:
4. Can you imagine why your treating doctor finds the appointments important?
  - Yes
  - No, because:
5. If I have complaints / symptoms / questions concerning my health I…
  - Go to my general practitioner first
  - Book an extra appointment at the bariatric department of the hospital
6. If you missed more than 1 appointment, what is the reason for this (multiple choice)?
  - I do not need any guidance / follow up
  - I have no complaints and so no reason to come in
  - I do not understand the value of the appointment
  - I do not see any improvement of my complaints despite the appointments
  - I am physically not capable of coming to the hospital
  - Coming to the hospital takes too much time
  - I do not have a good relationship with my treating doctor
  - It costs me too much money
  - I can not get any time off of work for the appointments
  - I simply forgot
  - Other reason:
7. Do you currently have a job?
  - Yes
  - No but looking
  - No I was declared incapacitated
  - No I do not want to work
8. If you have a job:
  - I am working part-time
  - I am working full-time
9. What is your annual family income approximately?
  - <20.000 euro
  - 20.000-40.000 euro
  - >40.000 euro
  - I do not want to disclose this
10. How do you feel about your financial situation?
  - I struggle to have enough each month
  - It could be better but there is enough
  - I am content
11. Do you have a high or low risk insurance? *
  - High risk
  - Low risk
  * In the Netherlands health insurance is mandatory. Individuals are however free to choose from either a ‘High Risk’ or a ‘Low Risk’ package. Despite both packages covering the exact same treatment, individuals select their preference annually depending on their individual, personal and/or financial circumstances. A low risk package is typically more expensive as the monthly premiums are high. However given these high premiums, the financial risk to the insured party, in the event of a claim, is low. This is because of the low excess charges associated with this product. In contrast, a High Risk package is generally more affordable for the general public due to the lower monthly premiums. The potential pitfall here arises in the event of a claim whereby the excess costs are significantly higher depending on the claim. For example, a person opting for Low Risk cover may pay a monthly premium of €200 to their health insurer and in the event of a claim they may only be liable for the first €300-400 of costs. On the contrary; a person paying €100 for High Risk cover may be liable for the first €700-800 for the same treatment.
12. Is your follow up appointment paid for by the health insurer or is it included in your excess costs?
  - Paid by the health insurer
  - Included in my excess costs
13. Do you currently have a partner?
  - Yes I am married
  - Yes we are living together
  - Yes but not living together
  - No
  - No, I am divorced
14. Do you have children?
  - Yes, all under 18 years old
  - Yes, all above 18 years old
  - No, I am pregnant
  - No, I want to have children
  - No, I do not want to have children
15. If you have any children, how many?
  - 1
  - 2
  - 3
  - >3
16. What is the highest certified education you have finished? *
  - Primary school
  - Mavo
  - Havo
  - VWO
  - HBO
  - University
  * Based on the Dutch university / scholar system with answer A being the lowest form of education and answer F being the highest
17. Do you drink alcohol?
  - Yes, 1-2 units a day
  - Yes, more than 2 units a day
  - Yes, 1-2 units a week
  - Yes, less than 1 unit a week
  - No
18. Do you smoke cigarettes?
  - Yes, 1-8 cigarettes a day
  - Yes, 8-19 cigarettes a day
  - Yes, more than a package a day
  - No
19. What is your average travel time to the follow up appointments?
  - Less than half an hour
  - 30-60 minutes
  - 1-2 hours
  - Over 2 hours
20. What is your mode of transportation to and from the follow up appointments?
  - I drive my car there
  - I ride my bike or I walk there
  - I come by public transport
  - Someone has to take me
21. What is your ethnic background?
  - Dutch
  - Turkish
  - Moroccan
  - Another country within Europe:
  - Another country outside of Europe:
22. Do you use medication for psychiatric complaints or disorders?
  - Yes
  - No
23. If you use medication for psychiatric complaints or disorders, which?
  - Depression
  - Anxiety
  - Sleeping disorders
  - Other:
24. Do you use strong painkillers such as morphine or its derivatives?
  - Yes, because I suffer from ….
  - No
25. I prefer my follow up appointments at:
  - The hospital because:
  - The general practitioner because:

This is the end of the questionnaire. Thank you so much for your cooperation.
